# Ainhum

**Published:** 1889-10-12

**Authors:** 


### AINHUM.

Mr. Gaynes Doyle, surgeon to the San Fernando Hospital, Trinidad, has recorded a case of "Ainhum." The disease commenced with shooting burning pains in the left little toe, on which a scab formed, which being removed left a narrow depression running partly round the toe. On this another scab formed, and that being removed the narrow depression was found increased. As the disease advanced the constriction became more marked, gradually narrowing the pedicle so that the little toe had the appearance of being attached by a ligature. The little toe was removed at the pedicle, and "the man made a good recovery." In the present case it is stated there was no history of either leprosy or syphilis. This curious malady has been supposed to be peculiar to the African race, which is incorrect, as it occurs to various other natives of Eastern countries. There are some points in the progress of the malady which are not mentioned in Mr. Doyle's case. The distal portion of the toe generally swells, and the pedicle of attachment is only brought into view by separating the walls of the furrow. The furrow is caused by a constricting band or a local *sclero-derma*. The cause is obscure, but Sir William Moore, in his "Manual of the Diseases of India," believes the condition may result as a manifestation of leprosy.

